# Salt Sensitivity: Causes, Consequences, and Recent Advances

**DOI:** 10.1161/HYPERTENSIONAHA.123.17959

**Published:** 2023-09-18

**Authors:** Matthew A. Bailey, Neeraj Dhaun

**Affiliations:** Edinburgh Kidney, University/BHF Centre for Cardiovascular Science, The Queen’s Medical Research Institute, University of Edinburgh, United Kingdom (M.A.B., N.D.).; Department of Renal Medicine, Royal Infirmary of Edinburgh, United Kingdom (N.D.).

**Keywords:** glucocorticoids, hypertension, immunity, inflammation, microbiota, potassium, sodium

## Abstract

Salt (sodium chloride) is an essential nutrient required to maintain physiological functions. However, for most people, daily salt intake far exceeds their physiological need and is habitually greater than recommended upper thresholds. Excess salt intake leads to elevation in blood pressure which drives cardiovascular morbidity and mortality. Indeed, excessive salt intake is estimated to be responsible for ≈5 million deaths per year globally. For approximately one-third of otherwise healthy individuals (and >50% of those with hypertension), the effect of salt intake on blood pressure elevation is exaggerated; such people are categorized as salt sensitive and salt sensitivity of blood pressure is considered an independent risk factor for cardiovascular disease and death. The prevalence of salt sensitivity is higher in women than in men and, in both, increases with age. This narrative review considers the foundational concepts of salt sensitivity and the underlying effector systems that cause salt sensitivity. We also consider recent updates in preclinical and clinical research that are revealing new modifying factors that determine the blood pressure response to high salt intake.

For most people, daily salt (sodium chloride) intake habitually exceeds recommended limits. This is true globally regardless of sex, age (including children), ethnicity, and socioeconomic status. The negative effects are long recognized: Huang Di’s *Neijing Suwen*, written c200 BCE, cautions that “if large amounts of salt are taken, the pulse will stiffen and harden.”^1^ The adverse effects of high salt intake are supported by clinical trials (see Supplemental Material) and are best evidenced for blood pressure (BP).^2^ Reducing salt intake toward recommended upper limits remains an important public health goal for many countries. The magnitude of the BP reduction would vary between individuals but population health gains would be made because cardiovascular risk falls with every mm Hg.^3^ A complementary precision strategy is to identify individuals who would most benefit from dietary salt-reduction, that is, those who are salt sensitive. This review will highlight key work that shaped the concept of salt sensitivity and its importance for health; provide a focused view on the causative physiological pathways and key modifying factors; and consider how knowledge of salt-sensitivity may be leveraged to improve human health.

## SALT INTAKE AND BP: A BRIEF HISTORY

A study published in 1904 showed that a dietary regimen of salt-free bread, meat, and bouillon reduced BP in 6 men and 2 women with hypertension.^4^ The investigators concluded that BP related directly to salt intake, attributed to retention of chloride because sodium was not yet measurable in laboratories. An insightful observation was that patients were either able to “accommodate themselves to the chloride saturation of their organism” or were not, that is, those in whom “chloride saturation is revealed by permanent hypertension.” Studies in the 1940s, using Kempner’s strict rice fruit diet also note the differential response to salt restriction: “in 2 of the 6 patients the BP declined to essentially normal levels and promptly rose again to the pretreatment values when 20 g of sodium chloride was added daily.”^5^ In these early studies, the concept of salt sensitivity and salt resistance is born.

Dahl and Love made the first at-scale (n=873) association between measured BP and a qualitative assessment of salt intake, noting that hypertension (BP>140/90 mm Hg) occurred more frequently in individuals self-declaring high salt consumption than in those who never added salt to food.^6^ This prompted the development through selective breeding of a rat model where BP was either salt sensitive or salt resistant.^7^ The Dahl salt–sensitive rat has been a cornerstone of research on the genetic and physiological mechanisms through which high salt intake causes hypertension and organ injury. The BP response to increased salt intake between salt-sensitive rodents and their salt resistant controls is often very large and starkly divergent. This does not accurately reflect the human condition in which the response is a continuous variable. It has nevertheless been a convenient research tool to view the human phenotype as binary, allowing categorization of individuals as salt sensitive or salt resistant.

## MEASUREMENT AND PREVALENCE OF SALT SENSITIVITY

Investigators mostly use 1 of 2 approaches to identify salt-sensitivity, intervening either with diets of known low or high salt content for periods of several days or with diuretics/saline to rapidly contract/expand intravascular volume. The individual’s mean arterial BP is measured, and an arbitrary threshold difference (either absolute or percentage change) between the 2 intervention stages is applied to define salt sensitivity or salt resistance. Such studies consistently categorize ≈30% of healthy humans as salt sensitive. This may be higher in some ethnicities.^8^ It is more prevalent in women, regardless of menopausal status.^9–11^ Prevalence increases with age^12^ and with comorbidities that impair kidney and vascular function (eg, diabetes, hypertension, kidney disease).

These approaches have several limitations. They are resource-intensive and largely applicable only in a research setting. Consensus protocols are lacking. It is uncertain whether rapid intravascular manipulation captures the biology of salt sensitivity. Dietary protocols also suffer from lack of standardization regarding the salt content of diets, the order and duration of exposure, and subsequent washout periods between diets.^13^ Importantly, it is not certain that sensitivity to the BP-lowering effects of salt restriction and sensitivity to the BP-elevating effects of high salt intake are mechanistically the same. Moreover, the low salt intakes used are not realistically achievable outside of the research setting and are substantially lower than real-life diets recommended for most normotensive and hypertensive individuals

**Figure 1.**
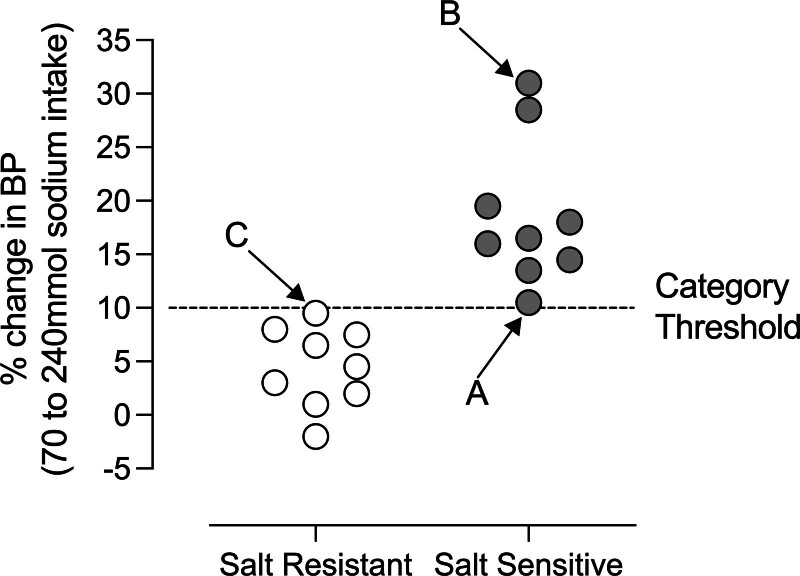
**Variability in the blood pressure (BP) response to salt.** The percentage change in mean arterial BP was measured in 18 hypertensives following a change from low to high salt intake, with an increase ≥10% as the category threshold. Subjects A and B are salt sensitive with changes of +10.5% and +31% respectively; subject C is salt resistant, with a change of +9.5%. The figure is illustrative of experimental data.^14^

The absence of standardized and evidenced category thresholds is problematic. Figure 1 shows categorization of 19 patients with hypertension.^14^ BP was measured after 6 days of a ≈0.6g/day salt regimen and after 6 days of ≈14g/day salt intake, with a category threshold of ≥10% increase in mean BP. Group-wise, the average BP response is clearly divergent, increasing by ≈18% in salt-sensitive and ≈4% in salt-resistant groups. However, given the spread of response across both groups, how likely is it that the 10% threshold is discriminating on core biological differences? Is salt-sensitive patient A more biologically aligned to salt-sensitive patient B or to salt-resistant patient C?

## SALT SENSITIVITY: WHAT DOES IT MEAN FOR CARDIOVASCULAR OUTCOMES?

BP is an accepted surrogate end point for cardiovascular and cerebrovascular events,^15^ but the connectivity between salt sensitivity and salt resistance defined in the research setting and the real-world BP of a given individual is not clear. The salt intakes deployed are extreme and the BP response is rapid, being achieved within days. This does not reflect real-world exposure and it is unlikely that individuals categorized as salt resistant in the research setting are impervious to the effects of a salt intake of 8 to 10 g/day sustained over decades, leading to accumulation in the skin and organ damage. This is an important consideration when communicating research outcomes. It is, however, likely that salt sensitivity captures important information concerning individual cardiovascular vulnerability. What that information means for hard health outcomes is not fully understood and our knowledge comes from just 2 retrospective studies from Japan^16^ and the United States.^17^

**Figure 2.**
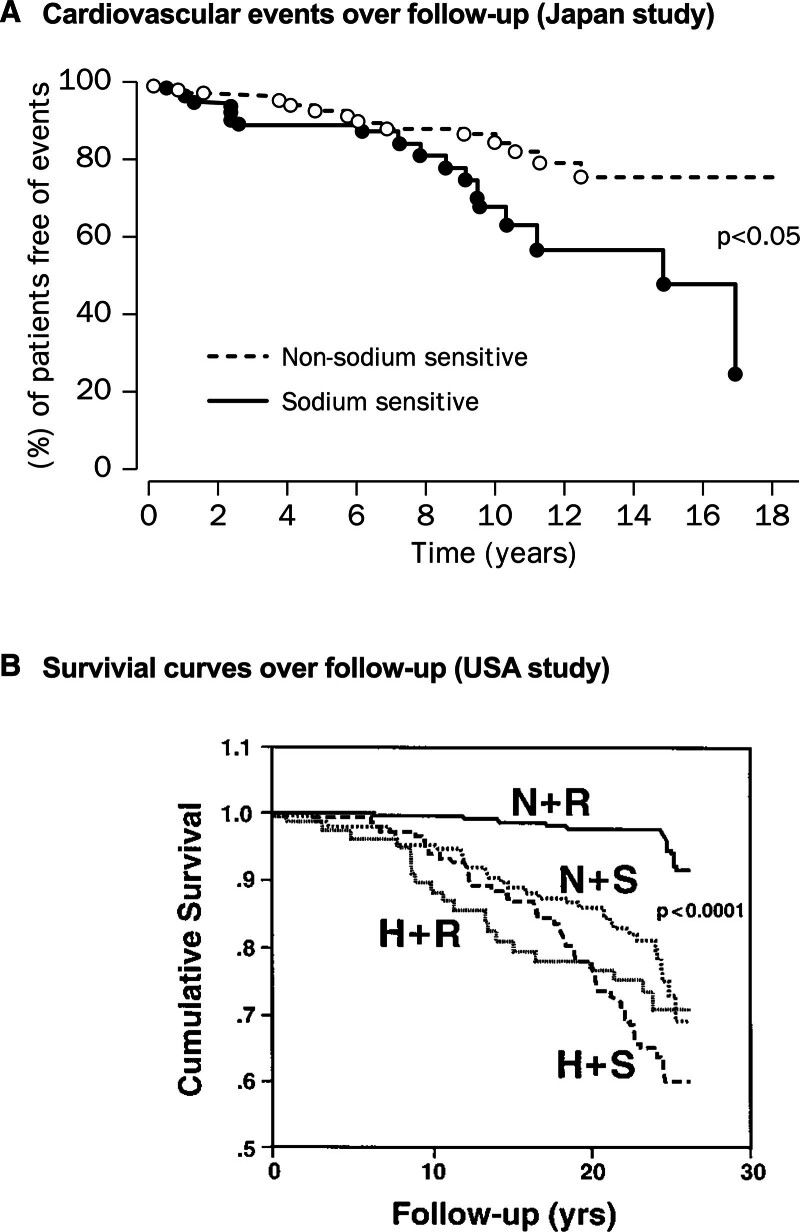
**The long-term impact of a salt-sensitive classification. A**, Study showing that salt-sensitive people had more cardiovascular events than nonsalt sensitive. In the salt-sensitive group, there were 17 (5 fatal) cardiovascular events and 14 (3 fatal) in the nonsalt-sensitive group. Reproduced from Morimoto et al^16^ with permission. Copyright ©1997, Elsevier. **B**, Survival curves for normotensive salt-resistant subjects (N+R), normotensive salt-sensitive subjects (N+S), hypertensive salt-resistant subjects (H+R), and hypertensive salt-sensitive subjects (N+S), Normotensive, salt-resistant people had a greater survival than those with hypertension and those with normotension and salt-sensitivity. Reproduced from Weinberger et al^17^ with permission. Copyright ©2001, Wolters Kluwer Health, Inc.

The Japanese study screened patients with essential hypertension for salt sensitivity using a dietary protocol following discontinuation of antihypertensive medication. Subjects were stabilized in hospital for ≈2 weeks and then a low (1–3 g/day) and high salt (12–15 g/day) diet for 1 week in a random order. BP and urinary sodium excretion were measured on the last 3 days of each diet period. Salt sensitivity was defined as a >10% increase in mean BP. After categorization, patients were managed in community to and objective BP of ≤140/90 mm Hg. Seven years later, records of 156 (62 salt sensitive) of the original ≈350 patients were screened for fatal and nonfatal cardiovascular events, kidney failure, and all-cause mortality. The calculated rate of total cardiovascular events per 100 patient-years in the salt-sensitive group was more than twice that of the nonsalt-sensitive group (Figure 2A). There were no cases of kidney failure reported in either group.

For the American study, subjects were admitted after antihypertensive withdrawal, and salt sensitivity was determined over 2 days. BP was initially measured before and after intravenous administration of 2 L of isotonic saline over a 4h period. The following day, subjects were sodium/volume depleted with 3 40 mg doses of furosemide and fed a low sodium diet (0.6 g/day salt), and BP was measured the following morning. Mean BP at the end of the saline infusion period was compared with that at the end of the furosemide/low-salt food-treatment, and salt sensitivity was defined as a difference of ≥10 mm Hg; salt resistance was defined as a difference <5 mm Hg and those with a BP change ≥5 and <10 mm Hg were categorized as indeterminate. Salt sensitivity was ascribed to 26% of the normotensive and 51% of the hypertensive subjects; 58% and 33%, respectively, were salt resistant at initial characterization.^18^ Over 25 years later, of the 596 subjects with available records, 123 had died, 60 from cardiovascular causes; these subjects were more likely to have been hypertensive and salt sensitive at the start of the study. The key point was that survival of subjects initially categorized as normotensive and salt sensitive was not different to those who were hypertensive. Normotension and salt resistance were associated with the best survival throughout follow-up (Figure 2B).

These studies are influential, with these limitations. The Japanese study, using a randomized dietary protocol that is highly reproducible in terms of categorizing an individual as salt sensitive or not,^13^ has a small number of subjects and short follow-up, resulting in few outcome events. The US study has important strengths in that it captured a higher proportion of the original cohort (≈85%) after a long follow-up. However, initial subject categorization rests on protocols that induce rapid intravascular volume expansion followed by rapid contraction, and the BP response to the contraction phase is arguably a form of furosemide testing, rather than sensitivity to salt intake per se. Both are retrospective studies with the inherent limitations that come from reviewing medical documentation that was not designed to collect data for research and having no control over subsequent exposure to salt intake or confounders. There are also risks of selection bias. For example, proportionally more of the 112 subjects lost to follow-up in the American study were young Black men.

The American Heart Association understands salt sensitivity as a cardiovascular risk factor “independent of and as powerful as BP.”^19^ We argue that the independence of salt sensitivity from BP, in terms of cardiovascular risk, remains unequivocally established. Given sustained high salt intake, salt-sensitive individuals may be vulnerable to higher BP in their day-to-day lives. Indeed, the Olivetti Heart study examined salt-sensitivity by dietary protocols in 47 normotensive men, and at 15-year follow-up (n=36), there was a higher incidence of hypertension in salt-sensitive subjects.^20^

## MECHANISMS OF SALT SENSITIVITY: EFFECTOR SYSTEMS AND MODIFYING FACTORS

Mean arterial BP is the product of cardiac output and total peripheral vascular resistance, with cardiac output being the product of heart rate and stoke volume. Salt sensitivity implies failure to adapt in ≥1 of these components. The 2 main theories have focussed on defects in (1) the renal regulation of intravascular volume and therefore cardiac output (renal dysfunction theory^21^), and (2) the regulation of vascular tone in resistance arteries and arterioles (vasodysfunction theory^22^).

### Renal Dysfunction

**Figure 3.**
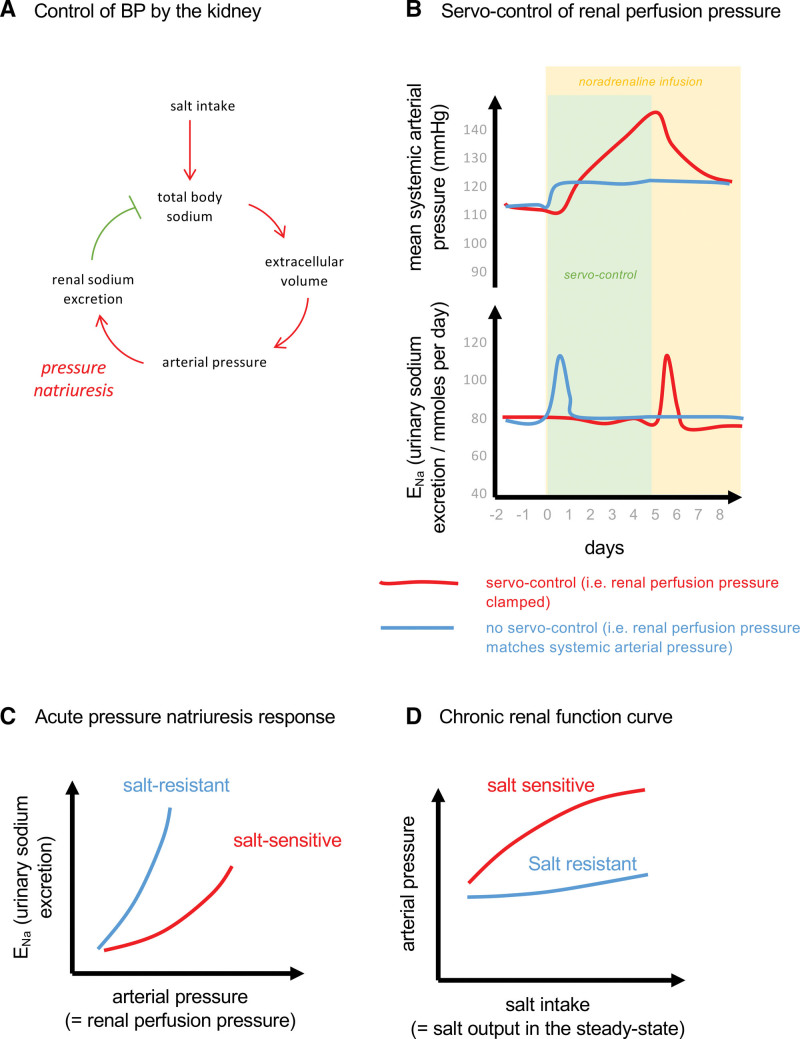
**Salt sensitivity, salt balance, and the kidney. A**, Negative feedback loop showing control of arterial blood pressure (BP) by renal sodium excretion. **B**, Servo-control of renal perfusion pressure highlights that the pressure natriuresis mechanism can influence chronic BP regulation. These experiments were performed in conscious dogs. In control animals (blue line), infusion of noradrenaline caused mean arterial pressure to increase by <10 mm Hg and an increase in sodium excretion. In animals with servo-controlled renal perfusion pressure, the BP response to noradrenaline was amplified and sodium excretion did not change. Removal of the servo-control allowed urinary sodium excretion to increase and BP normalized. The figure is illustrative of experimental data.^24^ **C**, The acute pressure natriuresis relationship may be attenuated in salt-sensitive individuals (red line) compared with nonsalt-sensitive people. The supporting evidence comes from studies in salt-sensitive animals, including studies in which major cardiovascular control systems have been experimentally disabled. **D**, The chronic renal function curve is often rotated and used to infer pressure natriuresis. In fact, the curve shows that salt-sensitive people cannot accommodate large changes in salt intake and BP rises.

Guyton et al’s^23^ hypothesis is that long-term regulation of arterial BP is achieved by the kidney. Every person has a set-point BP and any deviation from this causes a proportional change in renal artery perfusion pressure, triggering a parallel change in sodium excretion that will ultimately alter blood volume and cardiac output, returning BP to the set-point. High salt intake poses a physiological challenge: an increase in salt intake transiently expands plasma volume, increasing cardiac output; BP and renal perfusion pressure are increased, promoting sodium excretion, which in turn lowers effective blood volume and BP (Figure 3A). Central to this feedback loop is the physiological process of pressure natriuresis, in which increased arterial BP is sensed by the kidney as increased perfusion through the vasa recta, initiating paracrine signaling cascades leading to internalization of sodium transport proteins along the nephron.^25^ Evidence for the importance of pressure natriuresis for BP regulation comes from an experimental series in conscious dogs where an externally inflatable cuff was implanted around the aorta above the renal arteries, allowing investigators to control renal perfusion pressure independently of systemic arterial pressure.^24,26^ In one such experiment, noradrenaline was infused over a 7-day period (Figure 3B). When renal perfusion pressure was unregulated (ie, no external occlusion), noradrenaline increased mean arterial BP by 6-10 mm Hg; urinary sodium excretion initially doubled and dogs showed a negative cumulative sodium balance over the 7-day period; in contrast, when the cuff was used to maintain renal artery perfusion pressure within ≈1 mm Hg of baseline, noradrenaline infusion had no effect on sodium excretion and the BP rise was amplified, increasing by ≈35 mm Hg.^24^

The renal dysfunction theory proposes that salt sensitivity reflects a failure of the kidneys to excrete sufficient salt in response to an increase in salt intake, due to an underlying defect in the pressure natriuresis response.^16,27–29^ The idea that pressure natriuresis is impaired in salt-sensitive hypertension has been investigated in anesthetized animals: sodium excretion is measured during step-wise increases in renal perfusion pressure, typically achieved by sequential arterial ligation. The natriuretic response to increasing renal perfusion pressure, assessed over a short (minutes) timeframe, is attenuated in the Dahl salt–sensitive rat, compared with the salt-resistant strain^30^ (Figure 3C), and in mice models with salt-sensitive hypertension.^31^ Mechanistically, the blunted pressure natriuresis can be intrinsic to the kidney, reflecting, for example, failure of renal sodium transporters to downregulate with salt intake,^32^ abnormal paracrine signaling in response to rising perfusion pressure,^31^ or tubulointerstitial inflammation following immune cell infiltration.^33^ The intrinsic pressure–natriuresis relationship, which diminishes with age,^34^ is modulated by renal sympathetic nerve activity^35,36^ and by many endocrine factors, particularly the renin-angiotensin-aldosterone system.^37^

Nevertheless, such experiments do not test the central tenet of the renal dysfunction theory which holds that salt-sensitivity is caused by excess sodium retention secondary to impaired pressure natriuresis. In fact, despite attenuated pressure natriuresis, Dahl salt–sensitive display the same degree of sodium retention as Dahl salt–resistant rats when fed high salt.^38^ In other experimental settings of high salt intake, attenuated acute pressure natriuresis is observed without increased BP,^39,40^ and other studies show that with high salt intake, sodium balance can be achieved without increasing BP or renal perfusion pressure.^41^ Moreover, we are not aware of any studies that have directly measured the pressure natriuresis response in humans. Attempts have been made to infer the pressure natriuresis response from chronic renal function curves (Figure 3D). These curves are generated by directly changing salt intake or vascular volume and measuring BP as the dependent variable.^42^ Renal function curves are conventionally plotted with BP on the *x* axis and urinary sodium excretion on the *y* axis and may thus appear to show the chronic pressure natriuresis relationship. However, because both sodium excretion and BP are dependent on the independent intervention, which is not shown, renal function curves are difficult to interpret. The slope of the chronic renal function curve is attenuated in salt-sensitive individuals, but this does not show that the pressure natriuresis relationship is impaired. Rather, the curve highlights that those who are salt sensitive cannot accommodate large increases in salt intake and therefore BP rises. In summary, the acute pressure natriuresis response is suppressed in salt-sensitive animal models but whether this also occurs in humans and whether it is a causal phenomenon remain major unanswered questions.

### Vascular Dysfunction

**Figure 4.**
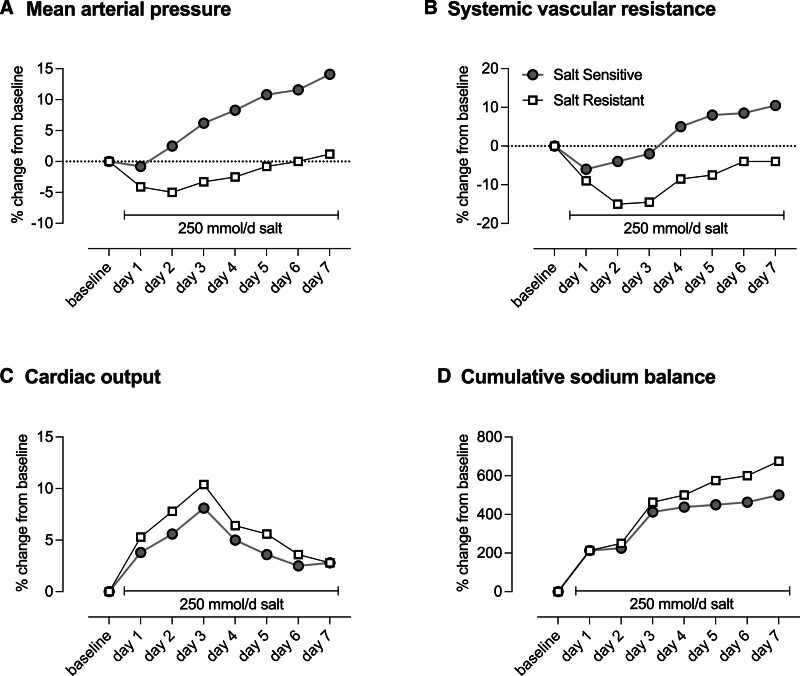
**Salt sensitivity and peripheral vascular resistance.** Effect of dietary salt loading (250 mmol salt per 70 kg per day) on (**A**) mean blood pressure (BP), (**B**) systemic vascular resistance, (**C**) cardiac output, and (**D**) cumulative sodium balance, shown as a change from baseline measurements (75 mmol/d salt per 70 kg day). Salt-sensitive individuals do not effectively reduce systemic vascular resistance in response to increasing salt intake. Salt sensitivity was not due to sodium retention and an exaggerated increase in cardiac output. The figure is illustrative of experimental data.^43^

Here, the concept is that an individual’s BP change in response to a dietary salt load is determined by their modulation of total peripheral vascular resistance, rather than a change in cardiac output (Figure 4). Most people respond with vasodilation and a fall in peripheral resistance; the salt load is accommodated and eventually excreted without inducing an exaggerated rise in BP. Salt-sensitive individuals, in contrast, display an abnormal vascular response: dietary salt-induced vasodilation is blunted, peripheral resistance does not fall, and the salt load induces a large rise in BP.

Key evidence for this theory comes from controlled clinical studies in healthy Black participants.^43,44^ During the first week, subjects were fed a low-salt diet (75 mmol Na^+^/day per 70 kg), increased to 250 mmol/day per 70 kg in the second week. BP was measured on the last 3 days of each period, averaged and if the difference between week 2 and week 1 was ≥5 mm Hg, participants were categorized as salt sensitive; <5 mm Hg denoted salt resistance. In the ≈50% of subjects who were salt resistant, the increased salt intake prompted a rapid drop in total peripheral vascular resistance, with a nadir of ≈15% at day 2. Peripheral vascular resistance progressively returned to presalt baseline by day 6. In salt-sensitive subjects, the initial fall in vascular resistance was blunted and—importantly—was not sustained, rising above baseline 3 to 4 days after the start of high salt intake. Cumulative sodium retention, increase in body weight, fall in hematocrit (indicating plasma volume expansion), and increase in cardiac output were similar between salt-sensitive and salt-resistant subjects.

The vasodysfunction theory acknowledges that increasing salt intake leads to short-term salt retention as the kidney takes several days to reduce tubular reabsorption and excrete the sodium load. However, the argument is that volume expansion and increased cardiac output occur with high salt intake, regardless of whether the individual is salt sensitive or not. Thus, volume expansion does not determine the BP response to a dietary salt load. Instead, it is a failure of the peripheral vasculature to rapidly dilate that causes salt sensitivity. Similar findings come from experiments in the Dahl rat. Changing sodium intake from 0 to 20 mmol/day caused hematocrit to fall and body weight to rise, consistent with plasma volume expansion and a gain in total body water, respectively. These volume effects were observed in both the Dahl salt–sensitive rats and nonsalt-sensitive control animals, with no differences between the 2; however, in the former, salt intake induced hypertension, and this was attributed to a failure of peripheral vascular resistance to fall as salt intake increased.^45^

Mechanistically, the hemodynamic dysfunction could be intrinsic to the arterial vasculature: defects in nitric oxide production by the endothelial cell^46^ and abnormalities in soluble guanylate cyclase signaling in the vascular smooth muscle cell^47^ are both reported. Nevertheless, many factors impact vascular resistance and the list of modifying factors that respond abnormally in a salt-sensitive paradigm include the sympathetic nervous system, endocrine factors, such as the renin-angiotensin-aldosterone system, and the milieu of paracrine agents (eg, endothelin-1) that can influence both endothelial and vascular smooth muscle cell biology.

### Unifying Mechanism for Salt Sensitivity?

Although desirable to attribute salt sensitivity to a unifying mechanism, we feel that current experimental evidence does not allow this. This partly reflects the technical challenges of assessing the function of all relevant physiological systems in any given experimental setting. Additionally, it remains unclear whether causal mechanisms established in one context (eg, salt sensitivity in health) are as important when transferred to another (eg, salt sensitivity in hypertension). Our own studies (Supplemental Material), suggest salt sensitivity can originate from direct defects in one or both effector systems. It may also arise due to an abnormality, congenital, or acquired in the array of factors that modify the function of these effector systems. Of these modifying factors, the sympathetic nervous system^48^ and renin-angiotensin-aldosterone system^49^ are long-established as major contributors to salt sensitivity. Indeed, the contribution of primary hyperaldosteronism and renin-independent aldosterone production is often underestimated and it may be one of the most common causes of salt sensitivity.^50^ In normotensives, renin-independent aldosteronism ranges across a continuum.^51^ This spectrum of renin-independent aldosterone secretion induces predictable alterations in renal sodium and potassium handling,^51^ but modeling in unilaterally nephrectomized rats shows that aldosterone initiates salt sensitivity by increasing total peripheral vascular resistance; cardiac output was reduced with high salt intake.^52^

## RECENT ADVANCES IN MODIFIERS OF SALT SENSITIVITY

In the remainder of this review, we will focus on recent advances highlighting the importance of other modifiers of salt sensitivity.

### Extracellular Potassium

The body contains ≈3.5 kg of potassium, mostly stored intracellularly, and extracellular potassium has a narrow physiological range, of 3.5 to 5.0 mmol/L. Chronic perturbations outside this range disturb the membrane potential of excitable cells and may be life-threatening.^53^

Potassium intake routinely falls below recommended adequate intake (90–120 mmol/day), and there is a negative association between potassium intake and BP, cardiovascular,^54–56^ and kidney disease.^57^ Interventional studies replacing regular table salt (100% NaCl) with low salt (75% NaCl, 25% KCl), show a reduction in BP,^58^ and meta-analysis of randomized controlled trials find that the hypotensive effect of oral potassium supplementation is larger in individuals with high sodium intakes.^59,60^ This leads to the hypothesis that subclinical potassium depletion contributes to salt sensitivity. Two lines of evidence support this hypothesis.

First, dietary potassium restriction, resulting in hypokalemia, induces salt sensitivity in young Sprague-Dawley rats.^61^ Second, clinical studies show that dietary potassium supplementation reduces BP to a greater extent in salt-sensitive individuals than in those who are salt insensitive, independent of sodium intake.^62^ In one study, normotensive individuals recruited from rural China underwent a sequential 3-stage protocol, eating first 3 g/day (51 mmol/d) NaCl, then 18 g/day (308 mmol/d) NaCl and then, in the final week, 18g/day NaCl with a 4.5g/day (60 mmol/d) KCl supplement. A BP response to salt loading of >10 mm Hg categorized 13 out of 60 participants as salt sensitive and potassium supplementation lowered their BP to the level recorded in the low-salt phase of the experiment. Mechanistically, salt-sensitive subjects had a lower 24-hour urinary sodium excretion than the nonsalt-sensitive group, a deficit abolished with potassium supplementation. Potassium salts have long been recognized for their diuretic potential and recent research has identified the underpinning molecular pathways in the renal tubule.^63^ Best understood is regulation of the NCC (sodium-chloride cotransporter) in the distal convoluted tubule, which normally reabsorbs ≈10% of the filtered sodium load and is the target of thiazide antihypertensives. Mutations in the key WNK4-SPAK-OSR1 (WNK Lysine Deficient Protein Kinase 4-Ste20-related proline alanine rich kinase-oxidative stress responsive kinase) cascade of regulatory kinases lead to gain of NCC function causing Gordon Syndrome (pseudohypoaldosteronism type II), which presents with salt-sensitive hypertension. Preclinical studies show that increasing plasma potassium by oral gavage of potassium-chloride promotes rapid dephosphorylation of serine and threonine residues in the N terminus of NCC and deactivates the transporter.^64,65^ The physiological rationale for this phenomenon is that by turning off NCC, high plasma potassium diverts sodium reabsorption from the distal convoluted tubule to the downstream collecting duct. Here, sodium is reabsorbed by the principal cell via the ENaC (epithelial sodium channel). ENaC-mediated reabsorption is electro-physiologically coupled to potassium secretion by ROMK (Reanl Outer Medullary K Channel) and BK (Big K) channels.^66^ Overall, the regulation of electrolyte transport in the kidney by plasma potassium means that potassium can be excreted without driving excess sodium reabsorption. Phosphoproteomic^67^ and transgenic mouse studies^68,69^ are unraveling the chain of events connecting a change in extracellular potassium to phosphorylation status of NCC. As extracellular potassium rises, a heterotrimeric potassium channel (Kir4.1/Kir5.1) is activated, depolarizing the basolateral membrane and reducing chloride efflux. Intracellular chloride concentration increases, inhibiting the phosphorylation of NCC by the WNK4-SPAK-OSR1 cascade.

The concept that dietary potassium supplements deactivate NCC has been validated in healthy, normotensive humans.^70^ Whether rapid, moment-to-moment control of NCC phosphorylation by plasma potassium influences BP is unclear. BP was not reduced by potassium supplements, although as indicated by the authors, the study was not powered to study BP changes.^70^ It is possible that deactivation of NCC by potassium improves the pressure natriuresis response, but this has not been tested directly. In the longer term, continued exposure to high potassium intake triggers ubiquitylation and lysosomal degradation of NCC, reducing the total NCC protein in the mouse kidney.^71^ This might be expected to facilitate sodium excretion and improve overall sodium balance, particularly in the setting of high sodium intake.

In salt-sensitive people, potassium supplementation also reduced plasma asymmetrical dimethylarginine, an endogenous inhibitor of nitric oxide synthesis, and increased urinary nitrite/nitrate excretion in salt-sensitive subjects.^62^ These effects are consistent with an increase in nitric oxide bioavailability and correction of a vascular defect by elevated potassium intake. Notably, raising potassium intake prevents the high salt–induced reduction of flow-mediated dilation in the brachial artery^72^ In rats, dietary potassium supplementation reduces salt-sensitive hypertension by inducing vasorelaxation, whereas potassium depletion creates a proconstrictive environment by stimulating the production of angiotensin II and endothelin-1, and reducing nitric oxide bioavailability.^73^ Cell culture experiments show that increasing extracellular potassium induces endothelial cell swelling and stimulates nitric oxide release; endothelial cell membrane stiffness is also reduced.^74^

Overall, dietary potassium emerges as an important modifying factor, and it is likely that modest, subclinical potassium depletion induces dysfunction in both the renal and vascular response to elevated salt intake. Extracellular potassium impacts other modulators of salt sensitivity, for example, T-cell function,^75^ which contributes to the injurious effects of high salt, as discussed below. Modifying dietary potassium to rescue salt-sensitivity may be feasible in certain settings.^76^ Hyperkalemia is a safety concern for use of oral potassium supplementation in certain patient groups,^53^ although potassium-rich diets seem well-tolerated in patients with advanced chronic kidney disease.^77^

### Glucocorticoids

Cortisol (corticosterone in rodents) production in the adrenal zona fasciculata is controlled by the hypothalamic-pituitary-adrenal axis (HPAA) and is not typically considered a salt balance hormone. Nevertheless, glucocorticoid excess (eg, Cushing syndrome) often causes salt-sensitive hypertension,^78^ as does glucocorticoid resistance (eg, loss-of-function mutations in the glucocorticoid receptor).^79,80^ Salt sensitivity in these different conditions relates either to hyperactivity of the HPAA and renin-angiotensin-aldosterone system or to abnormalities in the 11β-hydroxysteroid dehydrogenase enzymes that determine the level of active glucocorticoid in peripheral tissues.^78^ These important endocrine systems fail to adjust with salt intake and BP is responsive to mineralocorticoid receptor antagonists and glucocorticoid receptor antagonists. It is not surprising, given the powerful regulatory effects of these 2 systems, that salt sensitivity is associated with abnormalities in distal nephron sodium handling^81–83^ and vascular injury with hemodynamic dysfunction.^81,83–85^

Studies in humans and rodents also show connectivity between salt intake and HPAA function. There is a positive correlation between sodium excretion and urinary free cortisol excretion.^86,87^ Dietary interventional studies find that urine glucocorticoid excretion increases with salt intake.^88–90^ In C57BL6 mice, high salt intake causes multi-level disruptions in glucocorticoid biology,^91^ activating the HPAA and reducing corticosterone binding globulin. Overall, basal glucocorticoid levels and tissue exposure are enhanced by high salt intake. C57BL6 mice are often considered salt resistant,^19^ and salt-induced HPAA activation may exaggerated in salt-sensitive models.^92^

High salt intake amplifies stress-induced activation of the HPAA.^91^ In 1 study of 48 healthy normotensive White men, the HPAA response to acute mental stress was significantly greater in salt-sensitive subjects than those categorized as salt resistant.^93^ Chronic stress also induces salt sensitivity in young, normotensive subjects.^94^ The interplay between salt intake, basal cortisol, and the stress response is relevant to many contemporary lifestyles. An additional dimension comes from research in salt-sensitive rats in increased glucocorticoid and BP reflected salt induced modulation of the gut microbiota: reintroduction of intestinal *Bacteroides fragilis* inhibited the production of intestinal-derived corticosterone, mediated by bacterially derived arachidonic acid.^95^

### Gut Bacteria

The human body is colonized by large numbers of microorganisms (fungi, viruses, bacteria), mostly in the intestine where they support gut functionality through production of bioactive compounds, particularly short-chain fatty acids. There are >1000 bacteria species in the human gut, and biodiversity is affected by dietary constituents, including salt intake.^96^ Alterations in community structure are associated with, and may cause, pathophysiological changes to cardiovascular physiology.^97^

Our understanding of this emerging area mostly comes from animal research. Chronic angiotensin II infusion, a classic pharmacological model of salt sensitivity, decreases gut bacterial biodiversity, increasing the ratio of Firmicutes to Bacteroidetes, which are the major bacterial phyla. Disturbances in this ratio are found in many human bowel pathologies.^98^ In angiotensin II–dependent hypertension, the antibiotic minocycline rebalances the Firmicutes to Bacteroidetes ratio, reducing BP.^99^ Fecal matter transfer experiments show that the gut microbiome contributes to the salt-sensitive hypertension and renal injury in the Dahl salt–sensitive rat.^100^ CRISPR-Cas9 deletion of the Gper1 (G-protein–coupled estrogen receptor 1), protects against salt-sensitive hypertension in the Dahl rat. Particularly intriguing was the finding that genetic knockout of Gper1 strongly influenced the commensal bacteria colonizing the gut; this differed from wild-type rats although animals were maintained in the same environment, eating the same food.^101^ A fecal matter transplant from wild-type rats into the Gper1 knockout animals converted the gut microbiota signature of recipients and Gper1 knockout rats were no longer protected from salt-sensitive hypertension and endothelial dysfunction.^101^

Mechanistically, most work suggests that gut bacteria modulate the vascular effector system. Salt-induced alterations in the bacterial community and the agents produced by these colonies^102^ can induce vasoconstriction and amplify the BP response causing salt sensitivity. Short-chain fatty acids, such as acetate, and other byproducts of bacterial metabolism such as lactate, make important contributions to the levels circulating in the host serum. These are potent ligands of G-protein coupled receptors, such as GPR41^103^ and GPR81,^104^ and activation of these receptors constricts arteries, increasing peripheral resistance and BP.^101^

Gut bacteria can also influence the host vasculature indirectly by regulating the recruitment and polarization of immune cells. Thus, germ-free mice, born into and maintained in a sterile environment, have no commensal bacteria and are resistant to hypertension and vascular dysfunction induced by chronic angiotensin II. This protection arises because the recruitment of monocytes to the peripheral arterial vasculature that normally accompanies angiotensin II infusion is blunted in germ-free mice.^105^ Other studies examined fecal pellets from mice and found that high salt intake causes a rapid and sustained depletion *Lactobacillus murinus* from the gut and concomitant Th17 cell expansion. The growth of human and mouse *Lactobacillus* was inhibited by high extracellular sodium. Reintroduction of this bacterium into the gut microbiome repolarized Th17 cells and attenuated salt-sensitive hypertension.^106^ The modulation of immune cells by gut bacteria is mediated in large part by short-chain fatty acids.^107^

If salt sensitivity follows the gut microbiota, can this be leveraged to mitigate the adverse effects of high salt intake in some humans? Although an attractive hypothesis, the bacterial microbiome is highly complex to the extent that the community structure, and the activity of the bacterial bioactive pathways that influence host cell function, oscillate throughout the day, which may influence the diurnal BP rhythm.^108^ Moreover, broad-spectrum antibiotics, which reduce the diversity of the gut microbiome, amplify salt-sensitive hypertension in the Dahl rat but reduce BP in spontaneously hypertensive rats, which are less salt sensitive.^109^ The interplay between host strain genetics and different gut bacterial colonies makes it difficult to identify a common therapeutic strategy, although repurposing medicines used for disorders such as inflammatory bowel disease may show promise.^110^ Nevertheless, predicting the individual response to a therapy that alters the gut microbiome is a major translational roadblock.

### Immune Cells

Sustained high salt alters the activation state and profile of cells in both the innate and adaptive immune systems and research, mostly in animal models, shows that this contributes to salt-sensitive hypertension and tissue injury.^111^ For example, in the Dahl salt–sensitive rat, high salt intake causes an influx of macrophages, B and T cells into the kidney, a migration that does not occur in nonsalt-sensitive rat strains.^112^ Broad B- and T-cell suppression with mycophenolate mofetil attenuates hypertension in the Dahl salt–sensitive rat.^113^ Genetic depletion of CD3^+^ T cells is similarly protective^114^ and reconstitution of the T-cell population by adoptive splenocyte transfer restores salt-sensitive hypertension.^115^ This contributory role of adaptive immune cells to salt-sensitive hypertension is also evidenced in Rag1 knockout mice, genetically deficient in B and T cells, which have an attenuated BP response to angiotensin II.^116^

Recent advances reveal how high salt intake influences immune cells. Salt-induced increases in BP and renal perfusion pressure partially drive immune cell influx into the kidney.^117^ This is likely a responsive, secondary activation contributing to the progression of tissue injury. Other studies suggest a causal role in salt-induced hypertension itself. The gut microbiome releases short-chain fatty acids into circulation that impact the induction of innate and adaptive immune cells. If the normal host-microbiome interaction is disrupted by sustained high salt intake, the BP response to that salt is amplified.^107^ Moreover, innate and adaptive immune cells may be able to directly sense salt homeostasis, responding to extracellular sodium concentration via membrane channels and transporters. For example, increasing the concentration of NaCl in the extracellular media from 140 to 180 mmol/L activates p38/MAPK (p38 mitogen-activated protein kinases) pathways in human and mouse Th17 (T-helper 17)-cells, inducing proinflammatory polarization and augmenting production of TNF (tumor necrosis factor) α and interleukin-2.^118,119^ A similar tropic effect is seen on classically activated (proinflammatory) bone-marrow derived macrophages,^120^ whereas increasing extracellular salt concentration in vitro blunts the function of alternatively activated (anti-inflammatory) macrophages.^121^ In other studies, increasing extracellular sodium from 150 to 190 mmol/L promotes sodium entry into human dendritic cells, via ENaC and sodium-hydrogen exchanger isoform 1.^122^ This in turn increases intracellular calcium concentration due to calcium influx via the sodium-calcium exchanger. Protein kinase C is activated, phosphorylating p47phox and causing assembly of NADPH oxidase to drive superoxide and reactive oxygen species production. The oxidative burst has 2 effects: it directly activates the NLRP3 (NLR family pyrin domain-containing 3) inflammasome to produce interleukin-1β and it induces lipid peroxidation, which in the case of arachidonic acid, forms isolevuglandins that can act as neoantigens to activate T cells.^122,123^ Dendritic cells and other innate antigen-presenting cells orchestrate the immune balance between fighting invasive pathogens and the tolerance to self-antigens. As their function is influenced by salt intake, this has important consequences: clearly, the chain of events contributes to the development of salt-sensitive hypertension, from which NLRP3 deficient mice are protected.^123^ Longer term, the potential of antigen-presenting cells to hold a rapid immune memory to high salt is problematic and may contribute to tissue damage.

Translating these findings into the physiological setting of human salt homeostasis is the important next step. Salt intake is habitually high, and it is not yet known if the innate and adaptive immune systems operate differently in salt-sensitive and salt-resistant individuals, although cellular indexing of transcriptomes and epitopes sequencing (CITE-seq) of human monocytes suggests this to be the case.^123^ Moreover, how cells sense their ionic microenvironment in vivo is not resolved. Certainly, tissue interstitial sodium concentration is higher than that of plasma but only by ≈10 mmol/L.^124^ This may influence polarization and function of infiltrating and resident immune cells, consistent with the emerging view that such cells are involved in physiological sodium homeostasis. For example, monocyte-derived macrophages help the body meet the challenge of high salt intake by buffering the release of salt from the skin for renal excretion,^125^ or by promoting local vasodilation by scavenging the potent vasoconstrictor, endothelin-1.^126^ Disruption of either mechanism amplifies the salt-induced BP increase. In contrast, resident macrophages support physiological salt reabsorption by sustained sympathetic innervation of the kidney: disruption of this physiological crosstalk leads to natriuresis.^127^ Immune cells can also modify vascular function by inducing damage and fibrosis^128^ and disrupting endothelial cell integrity.^129,130^ An emerging concept is crosstalk talk between cells of the kidney tubule and immune cells. In models of type 2 diabetes, renal tubular cells secrete interleukin-1β, which induces proinflammatory polarization of macrophages and release of interleukin-6, to induce ENaC-mediated sodium retention and salt sensitivity.^131,132^ Activated T cells can interact with epithelial cells in the distal convoluted tubule, stimulating sodium transport through the thiazide-sensitive sodium-chloride cotransporter, salt retention, and high BP^133^; the inflammatory cytokine TNFα also has this effect.^134^

## CONCLUSIONS

We have aimed to identify the foundational basis for our understanding of salt-sensitive BP as an independent cardiovascular risk factor. Three things are clear: (1) salt sensitivity is a reproducible physiological phenotype that can be defined in a research setting using extreme changes in salt intake or intravascular volume; (2) thus defined, a large minority of healthy, normotensive people (and the majority of those with an underlying health condition such as kidney disease) have salt-sensitive BP; (3) there is a persistent environmental challenge of dietary salt excess, where intakes of 8 to 10 g per day are routine. High salt intake will exert a greater toll on some individuals than on others. Whether salt sensitivity increases cardiovascular risk independent of other risk factors, such as BP per se is less certain. Two clinical studies suggest this,^16,17^ but neither was designed to assess the BP load those participants experienced during the extended follow-up period. Barotrauma may not be the only cause of tissue injury by high salt intake.^135^ Animal research shows that high salt intake can impair many functions, including metabolism, immunity, and cognition. These studies should stimulate more research work in humans on potential adverse health effects of high dietary salt intake beyond BP.

Clinical research is hampered by the absence of standardized definitions and protocols to assess salt-sensitive BP. More problematic is the lack of a reliable and economic surrogate biomarker to take salt sensitivity out of the clinical research center and into real-world health care. This may come: RNA signatures of salt sensitivity in peripheral blood and other biofluids could pave the way to precision medicine by targeting nutritional therapy to those that will derive the most benefit.^136,137^

## ARTICLE INFORMATION

### Sources of Funding

The British Heart Foundation (FS/16/54/32730; FS/4yPhD/F/21/34166; PG/16/98/32568; RE/18/5/34216); Kidney Research UK (IN001/2017; INT001/2018), Chief Scientist Office Senior Clinical Research Fellowship (SCAF/19/02) and the Medical Research Council (MR/S01053X). For the purpose of open access, the authors have applied a Creative Commons Attribution (CC BY) license to any Author Accepted Manuscript version arising from this submission.

### Disclosures

The authors report consultancy fees from River2Renal (M.A. Bailey) and Travere Pharmaceuticals (N. Dhaun).

## Supplementary Material


