# Supplementary material for: Salt Sensitivity: Causes, Consequences, and Recent Advances
Source: Hypertension. 2023 Sep 18;81(3):476–89. doi: 10.1161/HYPERTENSIONAHA.123.17959 (PMC10863664; doi:10.1161/HYPERTENSIONAHA.123.17959)
Supplement: Supplementary file 1 [file hyp-81-476-s001.docx]

**Online Supplement**

**Salt sensitivity: causes, consequences & recent advances**

Matthew A. Bailey^1^

Neeraj Dhaun^1,2^

^1^*Edinburgh Kidney*, University/BHF Centre for Cardiovascular Science, The Queen's Medical Research Institute, University of Edinburgh, Edinburgh, UK.

^2^Department of Renal Medicine, Royal Infirmary of Edinburgh, Edinburgh, UK

Correspondence to: Matthew A Bailey, PhD FRSB

University/BHF Centre for Cardiovascular Science

Queen’s Medical Research Institute

47 Little France Crescent, Edinburgh.

E-mail: [matthew.bailey@ed.ac.uk](mailto:matthew.bailey@ed.ac.uk)

**Running title:** Advances in salt-sensitivity

**Keywords:** hypertension, potassium, inflammation, immunity, cortisol, glucocorticoid, microbiota

**Salt intake & blood pressure: evidence from clinical trials**

INTERSALT was the first systematic international observational study to assess the relationship between 24h urinary sodium excretion and BP, including >10,000 people aged 20-59 years across 52 centres.^1^ Protocols and practitioner training were standardized, and urinary electrolyte analysis was performed in a single laboratory. Using all 52 centres, INTERSALT found that urinary sodium excretion associated positively with median BP and hypertension prevalence. These associations were lost with exclusion of four centres of non-acculturated tribal populations with low sodium excretion (<51mmol/24h).^1^ An important finding was that the slope of increasing BP with age associated positively with sodium excretion. The overall conclusion from the INTERSALT investigators was that, at a population level, the effect of reducing salt intake on BP *per se* might be small but the impact on the age-related increase in BP, and by inference cardiovascular risk, is favourable.

Interventional trials of varying sizes and approaches have highlighted the benefits of reducing salt intake. The Trials of Hypertension Prevention (TOHP) studies, with a combined group size of 3,126 overweight but non-hypertensive participants, used nutritional counselling to reduce sodium intake to an objective target of ~1.6g/day (~4g/day salt) in the interventional arm, achieving a significant reduction in systolic BP.^2,3^ Incidence of hypertension was significantly reduced at 36 months and follow-up >10 years after the original trial found evidence for reduced cardiovascular risk.^4^

More recently, cluster randomized trials in Peru^5^ and China^6^ assessed the impact of community based salt-substitution strategies, replacing refined table salt (100% NaCl) with a 75% NaCl; 25% KCl mix (known as ‘low salt’). The Peruvian study assessed 2,376 people across 6 villages over 3 years, finding that the intervention reduced systolic BP by 1.3mmHg (-2.2 to -0.4) and diastolic BP by 0.8 (-1.4 to -0.1).^5^ Although these reductions may appear modest, at a population level, they would be estimated to reduce stroke and cardiovascular death incidence by 7-10%.^7^ Exploratory sub-analysis suggested that the salt-substitution intervention also significantly reduced the incidence of hypertension. The study from China (20,995 people across 600 villages) focussed on individuals at-risk of an adverse cardiovascular event: the population was elderly, 88% had a history of hypertension and >70% a history of stroke. Here, salt substitution reduced the rates of stroke, all cardiovascular events, and all-cause mortality during the ~5-year follow up period.^6^ Urinary electrolyte analysis suggested that salt-substitution reduced salt intake by <1g/day.^6^ Urinary potassium excretion was increased, and other studies, including the Dietary Approaches to Stop Hypertension (DASH) trial, suggest that a raised potassium intake can reduce BP independently of salt intake. DASH enrolled 459 pre-hypertensive adults and showed that a diet rich in fruit and vegetables and having reduced total/saturated fat decreased BP compared with a control diet reflecting typical American nutritional exposure, despite similar salt intakes of ~7.5g/day.^8^ The subsequent DASH-Sodium trial confirmed that salt intake reduction and the DASH diet reduced BP, with a greater effect if both were used in combination.^9^ We discuss the modifying effect of potassium on salt sensitivity later in this review.

To summarize, dietary salt intake is a clear risk factor for hypertension and cardiovascular disease. Salt intake, and therefore cardiovascular risk, is modifiable: managing intake is an attractive, and potentially cost-effective, means to improve health outcomes if it can be achieved safely at scale. There are significant complexities: the BP response to a given dietary salt exposure is a continuous variable, which means that some individuals would benefit more from dietary salt reduction than others.^10^ More problematic is the suggestion that reducing salt intake would increase BP in some individuals. Inverse salt sensitivity is report in clinical studies when salt intake is reduced to ~1g/day.^11^ The real-world relevance of the phenomenon warrants detailed investigation, being a potential safety barrier to population-level intervention. Identifying salt sensitive individuals within a population is the aspirational approach to precision management of cardiovascular risk.

**Mechanisms of Salt sensitivity**

Our own studies (**see Online Supplement**), suggest salt sensitivity can originate from direct defects in either the kidney or in the vasculature or within the many factors that modulate these effector systems. Taking our studies in experimental salt-sensitivity as an example, we find that increasing salt intake raises BP by ~10mmHg within days in young, healthy C57BL6 mice. This involves sympathetic activation and vascular mechanisms and there is no impairment of the renal pressure-natriuresis response.^12^ We also used the C57BL6 strain to model the human disorder of Apparent Mineralocorticoid Excess. This is caused by loss of function mutations in 11β hydroxysteroid dehydrogenase type 2 and presented with salt-sensitive hypertension. Our experiments in mice and rats show that renal salt retention now contributes significantly to salt-sensitivity in this context ^13^ ^14^ ^15^. Conditional knockout strategies have revealed additional complexities: deletion of 11β hydroxysteroid dehydrogenase type 2 in the nucleus of the solitary tract causes enhanced salt appetite and salt-sensitivity^16^; deletion in the renal tubule activates increases sodium retention and salt-sensitivity is prevented by diuretic therapy^17^ This multi-organ view of salt-sensitivity is observed in other models: salt sensitivity induced by pharmacological inhibition of nitric oxide synthase in rats reflects both a loss of vasodilation and high peripheral resistance as well as volume expansion with impaired pressure-natriuresis.^18^

**REFERENCES**

1. Intersalt: an international study of electrolyte excretion and blood pressure. Results for 24 hour urinary sodium and potassium excretion. Intersalt Cooperative Research Group. *BMJ*. 1988;297:319-328. doi: 10.1136/bmj.297.6644.319

2. The effects of nonpharmacologic interventions on blood pressure of persons with high normal levels. Results of the Trials of Hypertension Prevention, Phase I. *JAMA*. 1992;267:1213-1220. doi: 10.1001/jama.1992.03480090061028

3. Effects of weight loss and sodium reduction intervention on blood pressure and hypertension incidence in overweight people with high-normal blood pressure. The Trials of Hypertension Prevention, phase II. The Trials of Hypertension Prevention Collaborative Research Group. *Arch Intern Med*. 1997;157:657-667.

4. Cook NR, Cutler JA, Obarzanek E, Buring JE, Rexrode KM, Kumanyika SK, Appel LJ, Whelton PK. Long term effects of dietary sodium reduction on cardiovascular disease outcomes: observational follow-up of the trials of hypertension prevention (TOHP). *BMJ*. 2007;334:885-888. doi: 10.1136/bmj.39147.604896.55

5. Bernabe-Ortiz A, Sal YRVG, Ponce-Lucero V, Cardenas MK, Carrillo-Larco RM, Diez-Canseco F, Pesantes MA, Sacksteder KA, Gilman RH, Miranda JJ. Effect of salt substitution on community-wide blood pressure and hypertension incidence. *Nat Med*. 2020;26:374-378. doi: 10.1038/s41591-020-0754-2

6. Neal B, Wu Y, Feng X, Zhang R, Zhang Y, Shi J, Zhang J, Tian M, Huang L, Li Z, et al. Effect of Salt Substitution on Cardiovascular Events and Death. *N Engl J Med*. 2021;385:1067-1077. doi: 10.1056/NEJMoa2105675

7. Lewington S, Clarke R, Qizilbash N, Peto R, Collins R, Prospective Studies C. Age-specific relevance of usual blood pressure to vascular mortality: a meta-analysis of individual data for one million adults in 61 prospective studies. *Lancet*. 2002;360:1903-1913. doi: 10.1016/s0140-6736(02)11911-8

8. Appel LJ, Moore TJ, Obarzanek E, Vollmer WM, Svetkey LP, Sacks FM, Bray GA, Vogt TM, Cutler JA, Windhauser MM, et al. A clinical trial of the effects of dietary patterns on blood pressure. DASH Collaborative Research Group. *N Engl J Med*. 1997;336:1117-1124. doi: 10.1056/NEJM199704173361601

9. Sacks FM, Svetkey LP, Vollmer WM, Appel LJ, Bray GA, Harsha D, Obarzanek E, Conlin PR, Miller ER, 3rd, Simons-Morton DG, et al. Effects on blood pressure of reduced dietary sodium and the Dietary Approaches to Stop Hypertension (DASH) diet. DASH-Sodium Collaborative Research Group. *N Engl J Med*. 2001;344:3-10. doi: 10.1056/NEJM200101043440101

10. Kawasaki T, Delea CS, Bartter FC, Smith H. The effect of high-sodium and low-sodium intakes on blood pressure and other related variables in human subjects with idiopathic hypertension. *Am J Med*. 1978;64:193-198. doi: 10.1016/0002-9343(78)90045-1

11. Felder RA, Gildea JJ, Xu P, Yue W, Armando I, Carey RM, Jose PA. Inverse Salt Sensitivity of Blood Pressure: Mechanisms and Potential Relevance for Prevention of Cardiovascular Disease. *Curr Hypertens Rep*. 2022;24:361-374. doi: 10.1007/s11906-022-01201-9

12. Ralph AF, Grenier C, Costello HM, Stewart K, Ivy JR, Dhaun N, Bailey MA. Activation of the Sympathetic Nervous System Promotes Blood Pressure Salt-Sensitivity in C57BL6/J Mice. *Hypertension*. 2021;77:158-168. doi: 10.1161/HYPERTENSIONAHA.120.16186

13. Bailey MA, Craigie E, Livingstone DEW, Kotelevtsev YV, Al-Dujaili EAS, Kenyon CJ, Mullins JJ. Hsd11b2 haploinsufficiency in mice causes salt sensitivity of blood pressure. *Hypertension*. 2011;57:515-520. doi: 10.1161/HYPERTENSIONAHA.110.163782

14. Craigie E, Evans LC, Mullins JJ, Bailey MA. Failure to downregulate the epithelial sodium channel causes salt sensitivity in Hsd11b2 heterozygote mice. *Hypertension*. 2012;60:684-690. doi: 10.1161/HYPERTENSIONAHA.112.196410

15. Mullins LJ, Kenyon CJ, Bailey MA, Conway BR, Diaz ME, Mullins JJ. Mineralocorticoid excess or glucocorticoid insufficiency: renal and metabolic phenotypes in a rat Hsd11b2 knockout model. *Hypertension*. 2015;66:e20. doi: 10.1161/HYP.0000000000000035

16. Evans LC, Ivy JR, Wyrwoll C, McNairn JA, Menzies RI, Christensen TH, Al-Dujaili EA, Kenyon CJ, Mullins JJ, Seckl JR, et al. Conditional Deletion of Hsd11b2 in the Brain Causes Salt Appetite and Hypertension. *Circulation*. 2016;133:1360-1370. doi: 10.1161/CIRCULATIONAHA.115.019341

17. Ueda K, Nishimoto M, Hirohama D, Ayuzawa N, Kawarazaki W, Watanabe A, Shimosawa T, Loffing J, Zhang MZ, Marumo T, et al. Renal Dysfunction Induced by Kidney-Specific Gene Deletion of Hsd11b2 as a Primary Cause of Salt-Dependent Hypertension. *Hypertension*. 2017;70:111-118. doi: 10.1161/HYPERTENSIONAHA.116.08966

18. Wang C, Kawakami-Mori F, Kang L, Ayuzawa N, Ogura S, Koid SS, Reheman L, Yeerbolati A, Liu B, Yatomi Y, et al. Low-dose L-NAME induces salt sensitivity associated with sustained increased blood volume and sodium-chloride cotransporter activity in rodents. *Kidney Int*. 2020;98:1242-1252. doi: 10.1016/j.kint.2020.05.050
